# Changing Professional Behaviors in the Digital World Using the Medical Education e-Professionalism (MEeP) Framework—A Mixed Methods Multicentre Study

**DOI:** 10.3389/fmed.2022.846971

**Published:** 2022-03-28

**Authors:** Shaista Salman Guraya, Muhamad Saiful Bahri Yusoff, Fiza Rashid-Doubell, Denis W. Harkin, Suhail H. Al-Amad, Salim Fredericks, Mouhammad Omar O. Halabi, Natasya Abdullah, Hatem Moussa, Saad Imad Yousef Mallah, Jessica Atef Nassef Sefen, Heba Khalid A. Rahman Mohamed Ishaq AlKoheji, Manal Ebrahim Ali Althawadi, Lana Abdulsalam Alabbasi, Mohd Zarawi Mat Nor, Farida Reguig, Salman Yousuf Guraya

**Affiliations:** ^1^Royal College of Surgeons Ireland, Medical University of Bahrain, Busaiteen, Bahrain; ^2^Department of Medical Education, School of Medical Sciences, Universiti Sains Malaysia, Kelantan, Malaysia; ^3^Faculty of Medicine and Health Sciences, Royal College of Surgeons Ireland, University of Medicine and Health Sciences, Dublin, Ireland; ^4^College of Dental Medicine, University of Sharjah, Sharjah, United Arab Emirates; ^5^Faculty of Medicine and Health Sciences, Universiti Sains Islam Malaysia, Nilai, Malaysia; ^6^Department of Surgery, American Hospital Dubai, Dubai, United Arab Emirates; ^7^Clinical Sciences Department, College of Medicine, University of Sharjah, Sharjah, United Arab Emirates

**Keywords:** professional values, professional behaviors, professional identity, e-professionalism, digital world, Theory of Planned Behavior

## Abstract

**Background:**

There is increasing evidence on the exponential use of technology-based social media in medical field that has led to a proliferation of unprofessional behaviors in digital realm. Educating, training, and changing the behaviors of healthcare professionals are essential elements to restrain the rising unprofessional incidents. Therefore, this research was designed to determine the impact of an interventional workshop on the medical and dental students in improving their professional behaviors in the digital world using the newly developed medical Education e-Professionalism (MEeP) framework.

**Methods:**

We adopted the Theory of Planned Behavior (TPB) as a benchmark reference which explores constructs intertwined with the mission-based MEeP framework; values (whistleblowing-raising concerns), behaviors (being responsible in the digital world) and identity (reflective practice in the digital world). A multicentre 3-phased mixed-method study was conducted using a pre-workshop survey, an online interventional workshop, and a post-workshop survey. SPSS and NVivo were the tools used for the data analysis.

**Results:**

A total of 130 students registered for workshop out of which 120 completed the pre-workshop survey, 62 joined the workshop and 59 completed the workshop and post-workshop survey. From the *whistleblowing – raising concern* perspective, we found that attitudes and perceived behavioral control had a significant relationship. While for *responsible in digital world* category, attitude and perceived behavioral control had a significant bearing on the intentions. Third, for *reflective practice*, attitude and subjective norms significantly enhanced the intention of participants. A multi layered thematic analysis yielded four overarching themes of attitudes, subjective norms, perceived behavioral control and intentions. Most students showed positive *attitudes* of being *reflective, self-directed, and humane*. Students realized the *subjective norms* had made them *conscientious, self-aware and conformative*. While *perceived behavioural control* manifested as identity and *Intentions* were heavily reliant on *self-actualization*.

**Conclusion:**

Our mixed method study found that the interventional workshop using MEeP framework significantly improved attitudes, subjective norms, perceived behavioral control, and intentions. This study provides valuable evidence of MEeP framework evaluation using the theoretical underpinning of TPB by reporting positive changes in professional values, behaviors, and identities of undergraduate medical and dental students.

## Background

Medical professionalism is a multi-faceted construct which tends to foster professional values, behaviors and attitudes among medical students and healthcare professionals (HCPs) (1). The prime goal of medical professionalism remains a high-quality patientcare (2). Despite its sensitive nature and fundamental role in the medical sphere, we have witnessed lapses in medical professionalism which can potentially threaten patients' confidentiality, privacy, autonomy, and other core principles of medical ethics (3, 4). The most commonly reported domains of the lapses in professional behaviors have been rightly grouped into four Is; lapses in involvement, integrity, interaction, and introspection (5). These lapses can be partly attributed to the absence of a standard curriculum of medical professionalism worldwide (6).

Reciprocally, in concordance with the rapidly evolving technology based social networking sites, there is a growing interest in medical e-professionalism, “the behaviors and attitudes reflecting typical professionalism's examples that are manifested through social media” (7). Medical educators and policy makers look at e-professionalism as a crossroad between medical professionalism and social networking (8). Regrettably, there is a plethora of reports about the decay and gross violation of professional behaviors of medical students and physicians in the digital world (9, 10). These include, but are not limited to, unauthorized postings of patients' pictures, physician-patient communication podcasts, vlogs with obvious patient identification, HCPs' partying and drinking alcohol in inappropriate attires, and imparting inaccurate medical information mostly driven by commercial agenda of pharmaceutical companies (11).

Despite an abundance of lapses in e-professionalism in the medical field, little is known about the remedial efforts to rescue the prescribed codes of conduct in the digital world (3). Guraya et al., have recently developed a medical education e-professionalism (MEeP) framework; a mission-based social contract which furnishes essential competencies of e-professionalism for HCPs (12). The core tenet of MEeP framework provides a structured road map for a clear recognition of professional and personal digital space for HCPs in social media while preserving confidentiality, privacy, conscientiousness, accountability. The MEeP framework contains three major constructs of professional values (conformity, benevolence, universalism, and integrity), professional behaviors (communication, tolerance, and power) and professional identity formation (reflective, conscientious, self-directed, and self-actualization).

There is a little evidence about the use of a structured framework for an evaluation of the changes in professional behaviors and attitudes in the medical field. We employed the Theory of Planned Behavior (TPB) which examines a change in intentions by looking into attitudes, subjective norms, and perceived behavioral controls (13). TPB is a social psychological theory which has been widely used to predict human intentions which are precursor of behaviors. As described in TPB, attitudes refer to HCPs overall evaluation of behavior, subjective norms the degree of pressure felt from various organizations and people to act in a certain way, perceived behavioral control the confidence and beliefs of HCPs in their ability to carry out the behavior, while intentions are the extent to which HCPs intend to perform future professional behaviors. Keeping the enormous reach of digital world in mind where a single message/post/tweet can influence the life of millions. Competencies in the professional values construct of MEeP framework can be envisaged as an ability to “raise concerns” in case of any violation or breach of conduct (14). Continuous monitoring of users posts in terms of its nature, form and intent, social networking sites are generating digital algorithms (15). Such hidden manipulations have urged the need to depict responsible digital behaviors. So in the professional behavior construct “being responsible in digital world” was probed. The combination of digital power and manipulation has rendered the “self/identity” vulnerable. To mitigate the risks of this dynamic, intrusive, and manipulative world a reflective practice ensues a buffering effect (16). Therefore, there is a need to measure the intention for being reflective in digital world. In order to evaluate the MEeP framework, in this mixed-method study, we ascertained the changes in professional values, behaviors, and identities of the undergraduate medical and dental students during an interventional workshop.

## Materials and Methods

In this mixed-method funded research project, we evaluated the MEeP framework in determining the change in professional values (whistleblowing-raising concerns), behaviors (being responsible in digital world) and identity (being reflective in digital world) of the undergraduate medical students of Royal College of Surgeons Ireland (RCSI), Bahrain, Medical University of Bahrain (RCSI-MUB) Bahrain, University Sains Islamic Malaysia (USIM) Malaysia and undergraduate medical and dental students from University of Sharjah (UoS), United Arab Emirates (UAE). This study used an interventional workshop for determining the changes in professional values, behaviors, and identities of the undergraduate medical and dental students of three medical institutions. To achieve this objective, we evaluated these constructs using the concepts of whistleblowing-raising concerns (professional values), being responsible in the digital world (professional behavior) and being reflective in the digital world (professional identity), respectively.

The following research questions were used in our research.

1) Does the MEeP framework improve the understanding of the expected competencies in the digital world?
2) Will the MEeP framework interventional workshop improve the professional attitudes and behaviors of the participants about whistleblowing, reflective practice and being responsible in the digital world using TPB?

### Research Design

During August 2021, we conducted our mixed-method study, a pre-workshop survey, an interventional workshop and a post-workshop survey. We replicated a pragmatic study strategy used by Guraya et al. (12) keeping axiology, epistemology and ontological perspectives in mind (17). An email invitation was sent to all undergraduate medical and dental students of RCSI-MUB), USIM, and UoS). The invitation included details of the research study, a participant information leaflet, and a consent form. A unique participant ID was sent only to students who expressed an interest to participate in the research. A week before the workshop, a link was then sent to all registered participants via SurveyMonkey which contained TPB questionnaire (Appendix I). The participants who completed the pre-workshop survey received another link containing three pre-recorded lectures about an introduction to e-professionalism, details of the MEeP framework, and information about case scenario learning. Additionally, an invite to participate in the interventional workshop was also included. A 2-h online structured workshop was conducted with the help of trained facilitators. A dedicated facilitators guide and toolkit was prepared along with numerous meetings to standardize the facilitation process A comprehensive plan of the interventional workshop is enclosed in Appendix II. Finally, a post-workshop survey was administered using the same TPB questionnaire immediately after the participants had completed the workshop.

### Ethical Approval

Approval for the study was obtained from 3 different universities in three different countries.

Royal College of Surgeons Ireland – Bahrain REC **139/25-Mar-2021**

University of Sharjah **REC-21-06-03-01**

University Sains Malaysia Research Ethics Committee **(JEPeM) USM/JEPeM/19050291**.

### Quantitative Methods

Using a convenient sampling design, we sent an online invitation was sent to all the registered undergraduate medical students across all years in the participating institutions. There are varied recommendation depending upon the research context, we did not stipulate a minimum sample size requirement (18). The quantitative data from pre-and post- workshop surveys was collected and analyzed using the Statistical Package for Social Sciences (SPSS) 24.0 software. We adopted a TPB questionnaire from the study by Medisauskaite et al. (19) (Appendix I). The TPB questionnaire collected demographic information of age, gender, ethnicity, place and year of study, and students' previous exposure to professionalism teaching. Attitudes, subjective norms, perceived behavioral control, and intentions were measured on a 7-point Likert scale that ranged from 1 to 7. Higher scores indicated more positive attitudes, norms, perceived controls, and intentions. We measured the internal consistency of the scales of changing professional behaviors in digital world. An internal consistency of the instrument was measured by calculating Cronbach's alpha (cut-off *0.70*) (20).

In the univariate analysis, we used paired sample *t*-test for a comparison of responses to statements in the questionnaires that were administered before and after the workshop. In the descriptive statistics, we reported the mean, standard deviation, minimum and maximum values of scales. In the multivariate analysis, intentions were considered as dependent while attitudes, subjective norms and perceived behavioral control as independent variables. We employed a path analysis based on regression analysis of scales of changing professional behaviors. However, before the path analysis was carried out, we conducted a correlation analysis to ensure a linear relationship between variables to determine the possible chances of multicollinearity. A *p-*value of 0.05 or less was considered as significant. A structural equation modeling (SEM) was performed to assess the interrelations between observable variables in the retrieved data using the Analysis of Moment Structure (AMOS) software to determine the achievement of the minimal requirement of goodness of fit indices: where the acceptable values were considered as comparative fit index (CFI) more than 0.90 (21), goodness of fit index (GFI) more than 0.90 (22), Tucker–Lewis index (TLI) more than 0.90 (23), root mean square of error approximation (RMSEA) < 0.80 (24)and Chi-square/degree of freedom (χ^2^/df) < 3 (25–27).

### Intervention

The online interventional workshop consisted of a 2-h structured program using Zoom Video Communications, Inc. The data was collected from small groups (10–12 participants) using structured case-based discussions facilitated by a member of the research team. There were 15 facilitators, who were trained using the Zoom application, and training included outlining the facilitators' roles in breakout rooms, facilitating a mock session in a breakout room, practicing the functionality of a digital interactive whiteboard: Jamboard-Google and rehearsing how to supervise small group interactive sessions using technology. A comprehensive facilitator's guide was produced and distributed to all facilitators before the start of the training session (Appendix III). A trained IT support person was recruited to monitor and to provide technical support during all phases of our research.

During the workshop, in the main meeting room, the principal investigator introduced facilitators to all participants, and briefly described the objectives and perceived outcomes of the workshop. Participants were requested to abide by data privacy regulations and their institutional code of professional conduct. Afterwards, all participants were distributed to breakout rooms where, in the presence of trained facilitators, three to five hypothetical case scenarios were discussed. A total of eight hypothetical cases about the lapses of professional behaviors in the digital world were used to generate discussions and to attain plausible solutions using the MEeP framework as a reference. These cases were developed by all researchers using the three Ps model of Biggs; presage, process, and product (28). The final list of cases was approved after several rounds of reviews and discussions. We took inspiration from GMC and AAMC guidelines while constructing the cases (29–31). Table 1 illustrates the mapping of competencies and constructs of MEeP framework with the relevant core concepts of the selected eight cases in our study. During the breakout discussions by various groups, the key messages from each case were identified and documented on the Jamboard, a digital interactive whiteboard developed by Google. Later, all breakout room teams reconvened back to the main meeting room and a pre-nominated spokesperson from each team presented the group's key findings. Finally, moderators wrapped up the session and a post-workshop survey link was sent to all participants.

**Table 1:** Mapping of competencies and constructs of the MEeP framework with eight case scenarios selected the framework evaluation.

**No**.	**Case title**	**MEeP Framework competencies and constructs**
1.	Free speech vs. professionalism	Benevolence (Values)
		Power (Behavior)
		Self-direction (Identity)
2.	The never forgiving digital world!	Communication (Behavior)
		Self-actualization (Identity)
		Reflective (Identity)
3.	A medical student on vacation	Benevolence (Values)
		Integrity (Values)
		Reflective (Identity)
4.	This platform is strictly professional!	Conscientious (Identity)
		Communication (Behavior)
		Self-direction (Identity)
5.	Is anything ever private?	Power (Behavior)
		Self-direction (Identity)
		Self-actualization (Identity)
6.	WhatsApp is a closed space!	Conscientious (Identity)
		Integrity (Values)
		Conformity (Values)
7.	Mr. Google's wise opinion	Benevolence (Values)
		Conscientious (Identity)
		Integrity (Values)
8.	Social media saved my son!	Conscientious (Identity)
		Power (Behavior)
		Universalism (Values)
		Integrity (Values)

### Qualitative Methods

Participants were informed in detail of workshop proceedings in the Participant information leaflet. They provided consent to all the audio recordings and anonymous Jamboard contributions analysis. All audio recordings of the workshop were verbatim transcribed by one researcher using Trint software, while the Jamboard data was exported on word document. We followed the thematic analysis approach by Braun and Clarke (32), which contains a six-phase process to capture unique findings from the transcripts. However, the analytical process characteristics of each phase was not confined to that phase rather kept on moving back and forth to ensure a continued and prolonged engagement with the data. The analysis of case discussions focused on identifying TPB and MEeP constructs. In this study, we performed three layers of analysis. First, as a benchmark, we followed a deductive analytical approach for the TPB constructs; second, a deductive approach to analyse the MEeP framework findings; an inductive approach to ascertain unique findings that were not already detected by the deductive approach. All these phases were augmented by revisiting the literature to help inform analysis. Using multiple iterations, sensitized by the TPB constructs and MEeP framework attributes, a thematic analysis framework was developed. The emergent themes generated by merging codes helped us in the identification of patterns. Converging and diverging patterns were identified between two researchers using mind and concept maps. Two researchers SSG and MOH developed and coded scheme by independently analyzing one of the Jamboards and an audio transcript of the breakout rooms. Once the initial framework was agreed upon, the rest of coding was done using the thematic framework with NVivo 12 - QSR international software.

### Ensuring Research Rigor

In our research the credibility of information was maintained by combining quantitative and qualitative data (33). Thick descriptors were provided in the detailed methodology section as appendices (questionnaire, workshop structure and facilitator's guide). Despite data collection and analysis are presented in two different sections, we found a significant overlap of concept evolution. TPB and MEeP framework constructs guided the questionnaire selection and case construction. Numerous meetings to discuss the intervention structure, content and flow along with facilitators training sessions added rigor to the research process. While analyzing data, a constant comparative process was established to avoid over or underrepresentation of the findings ensuring research rigor. A deductive and inductive lens and constant revisiting of data generated some interesting findings in the intentions theme. To triangulate and ratify our results we were mindful of any counter or aberrant evidence indicating the researcher's reflexivity. The originality of raw data was preserved by saving the original Jam boards contents NVivo 12-QSR international software as transcribed files.

## Results

### Quantitative

An online invitation was sent to all the registered undergraduate medical students across all years in the participating institutions. A total of 130 students registered for the workshop, out of which 120 completed the pre-workshop survey, 62 joined the workshop and 59 completed the workshop and post-workshop survey. The demographic characteristics of 59 participants are shown in Table 2. All results reported below are extracted from 59 respondents who completed all phases of the interventional workshop and surveys.

**Table 2:** Demographic characteristics of our study participants (*n* = 59).

**Demographics**	**No. (%) of participants**
**Gender**	
Male	19 (32)
Female	40 (68)
**Level of Study**	
Pre-clinical	29 (49)
Clinical	30 (51)
**Exposure to professionalism teaching**	
Yes	38 (64)
No	21 (36)

To assess the attrition bias, responses from students who completed questionnaires at pre-post workshop were compared to those who only completed questionnaire in pre-workshop. We did not find significant differences among the responses about demographic variables such as age, gender, college, year of study, and professional behavioral changes. Table 3 shows the reliability of scales of changing professionalism behaviors variables in our study in post-workshop survey. We found that the Cronbach's alpha (α) values of all scales were greater than the cut-off point 0.70, which indicated the reliability of scales. The results of the univariate analysis of the study using paired sample *t*-test are shown in Table 4.

**Table 3:** Questionnaire reliability measure analysis in post-work-shop survey (*n* = 59).

**Measures**	**Scale**	**Cronbach**	**No of**
		**alpha**	**items**
Whistleblowing—Raising	Attitudes	0.766	6
concern	Subjective norms	0.92	11
	Perceived behavioral control	0.739	2
	Intentions	0.803	3
Responsible in	Attitudes	0.828	8
the digital world	Subjective norms	0.959	12
	Perceived behavioral control	0.737	4
	Intentions	0.854	3
Reflective	Attitudes	0.873	8
practice	Subjective norms	0.875	12
	Perceived behavioral control	n/a^a^	1
	Intentions	0.761	3

*Cronbach's alpha 0.7–0.79 acceptable, 0.8–0.89 good, 0.9 or higher excellent*.

a *Cronbach's alpha cannot be calculated for 1-item measures*.

**Table 4:** A univariate analysis of MEeP domain scores following an online social media professionalism intervention (*n* = 59).

**Measures**	**Scale**	**Average**		**Diff**	**St_Err**	**t_value**	***p*_value**
		**Post**	**Pre**				
Whistleblowing—Raising concern	Attitudes	5.654	5.095	0.559	0.123	4.550	0.000^*^
	Subjective norms	4.030	3.978	0.052	0.185	0.300	0.780
	Perceived behavioral control	5.664	5.043	0.621	0.171	3.650	0.001^*^
	Intentions	4.632	4.285	0.347	0.104	3.336	0.003^*^
Responsible in digital world	Attitudes	6.244	6.138	0.106	0.103	1.050	0.309
	Subjective norms	4.965	4.205	0.759	0.171	4.439	0.000^*^
	Perceived behavioral control	5.938	5.230	0.708	0.158	4.481	0.000^*^
	Intentions	6.551	6.449	0.103	0.101	1.050	0.263
Being reflective in digital world	Attitudes	6.392	5.934	0.458	0.097	4.750	0.000^*^
	Subjective norms	4.615	3.783	0.832	0.187	4.450	0.000^*^
	Perceived behavioral control	5.397	5.914	−0.517	0.162	3.191	0.009^*^
	Intentions	4.620	4.782	−0.161	0.102	1.600	0.118

* *p-value < 0.001*

*Variables were scored from 1 to 7 where 7 scored the highest except the statements which were reversely interpreted as shown in Appendix 1*.

In the *whistleblowing – raising concerns* perspective, we found that attitude, perceived behavioral control and intention of participants significantly improved in the post-workshop phase. For *responsible in digital world* category, we found that only two scales of subjective norms and perceived behavioral control significantly improved during the post-workshop stage. However, we did not find any difference in *attitude and intentions* of participants. Finally, for *reflective practice*, attitude, subjective norms, and perceived behavioral control of participants improved substantially by the workshop. However, intentions and reflections of students' behaviors remained unchanged during the pre-post workshop. In conclusion, we observed a significant improvement in the students' professional behaviors in the post-workshop analysis as reflected by a higher mean of all scales in the post-workshop survey. Therefore, we further focused on the data of the post-workshop analysis.

Table 5 lays down the results of the correlation analysis of the post-workshop data to examine the possible chances of multicollinearity in our study. We observed that a highest correlation between two independent variables did not exceed the cut-off point 0.70 with no chance of multicollinearity in this data. In Table 5, from the *whistleblowing – raising concern* perspective, the participants' attitudes had a positive and significant relationship with intentions (r = 0.537, *p* < 0.01). This indicates that a higher value of attitude leads to a greater intention, which ultimately enhances the professional behavior of participants. Likewise, we found that perceived behavioral control had a positive and significant relationship with intentions (r = 0.361, *p* < 0.01); a higher value of perceived behavioral control enhances intentions. For *responsible in digital world* category, similar results were reported,; attitudes (r = 0.605, *p* < 0.01) and perceived behavioral control (r = 0.484, *p* < 0.01) had a positive associations with intentions. In the *reflective practice* category, we found that attitudes (r = 0.521, *p* < 0.01) and subjective norms (r = 0.355, *p* < 0.01) significantly enhanced the intentions of participants.

**Table 5:** A correlation analysis of MEeP domain scores following an online social media professionalism intervention (*n* = 59).

**Measures**	**Scale**	**1**	**2**	**3**	**4**	**5**	**6**	**7**	**8**	**9**	**10**	**11**	**12**
Whistleblowing—raising concern	1. Attitudes	1											
	2. Subjective norms	−0.133	1										
	3. Perceived behavioral control	0.561^**^	−0.082	1									
	4. Intentions	0.537^**^	0.031	0.361^**^	1								
Being responsible in digital world	5. Attitudes	0.661^**^	0.032	0.391^**^	0.389^**^	1							
	6. Subjective norms	−0.003	0.551^**^	0.144	0.099	0.239	1						
	7. Perceived behavioral control	0.586^**^	0.026	0.487^**^	0.442^**^	0.587^**^	0.092	1					
	8. Intentions	0.545^**^	−0.112	0.534^**^	0.227	0.605^**^	0.124	0.484^**^	1				
Being reflective in digital world	9. Attitudes	0.689^**^	−0.01	0.405^**^	0.328^*^	0.578^**^	0.18	0.557^**^	0.639^**^	1			
	10. Subjective norms	0.068	0.578^**^	0.139	0.168	0.182	0.490^**^	0.149	0.097	0.142	1		
	11. Perceived behavioral control	0.269^*^	−0.122	0.385^**^	0.026	0.404^**^	−0.122	0.339^**^	0.415^**^	0.415^**^	−0.168	1	
	12. Intentions	0.516^**^	0.176	0.120	0.639^**^	0.513^**^	0.261^*^	0.481^**^	0.152	0.521^**^	0.355^**^	−0.078	1

*The correlation coefficient (r) values*

** *p < 0.01*;

* *p < 0.05, respectively*.

**Figure 1:**
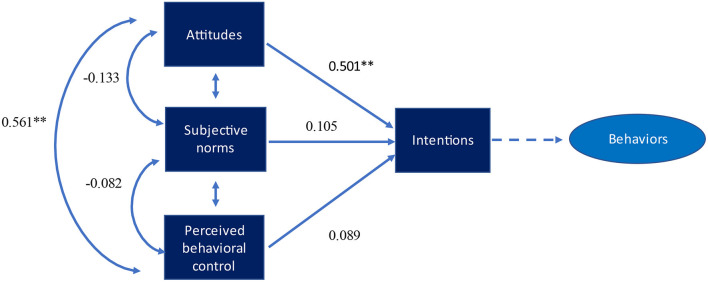
Path model with standardized regression coefficients for whistleblowing—raising concerns in digital world. *p < *0.05;* **p < *0.01*.

**Figure 2:**
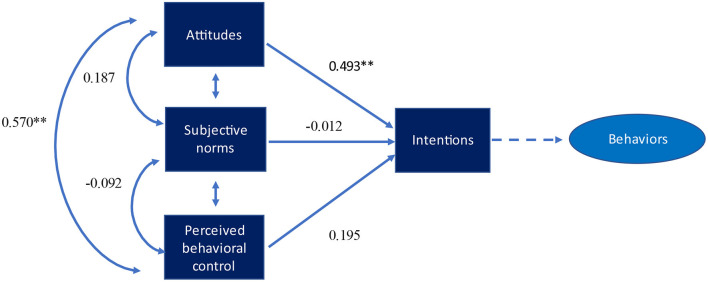
Path model with standardized regression coefficients for being responsible in digital world. *p < *0.05;* **p < *0.01*.

**Figure 3:**
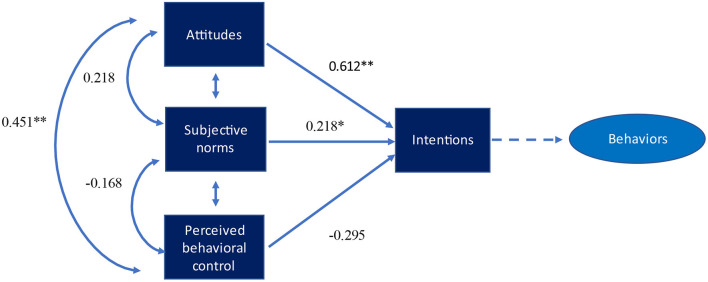
Path model with standardized regression coefficients for being reflective in digital world. *p < *0.05;* **p < *0.01*.

Figure 1 shows the path analysis of professional behavioral scales for *whistleblowing – raising* concern. The regression coefficient analysis revealed significant paths between intentions and perceived behavioral control (β = 0.561, *p* < 0.001) and intentions and attitudes (β = 0.501, *p* < 0.001). This shows that an increase in the value of attitudes leads to greater intentions which ultimately strengthen professional behavior. We also found significant regression coefficients paths between attitudes and perceived behavioral control (β = 0.561, *p* < 0.001). However, the path analysis didn't show a significant relationship between subjective norms and intentions, and perceived behavioral control and intentions. Figure 2 shows similar findings for *being responsible in the digital world*. The regression coefficients showed significant paths between attitudes and intentions (β = 0.493, *p* < 0.001), and attitudes and perceived behavioral control (β = 0.570, *p* < 0.001). Finally, Figure 3 *being reflective in the digital world* reported similar results as regression coefficients carried significant paths between attitudes and intentions (β = 0.612, *p* < 0.001), and attitudes and perceived behavioral control (β = 0.451, *p* < 0.001). Table 6 endorses the goodness of the fit of our study model as they exceeded the defined cut point values and validated the findings of our study. We evaluated the path model using several criteria with comparative fit index (CFI) values >0.90 and root mean square error of approximation (RMSEA) values of 0.80 or lower indicating a good fit of the model to the data (34).

**Table 6:** The goodness of fit indices used to signify model fitness (n=59).

**Fit indices**	**Rule of thumb**	**Raising concerns**	**Responsible in digital world**	**Reflective in digital world**
RMSEA	≤0.08	0.06	0.05	0.05
GFI	>0.90	0.92	0.91	0.94
CFI	>0.90	0.97	0.94	0.93
TLI	>0.90	0.93	0.92	0.96
χ^2^/df	<3	2.317	2.290	2.471

*χ2/df, Chi-square/degree of freedom; RMSEA, root mean square of error approximation; GFI, goodness of fit index; CFI, comparative fit index; NFI, normed fit index; TLI, Tucker–Lewis index*.

### Qualitative

**Figure 4:**
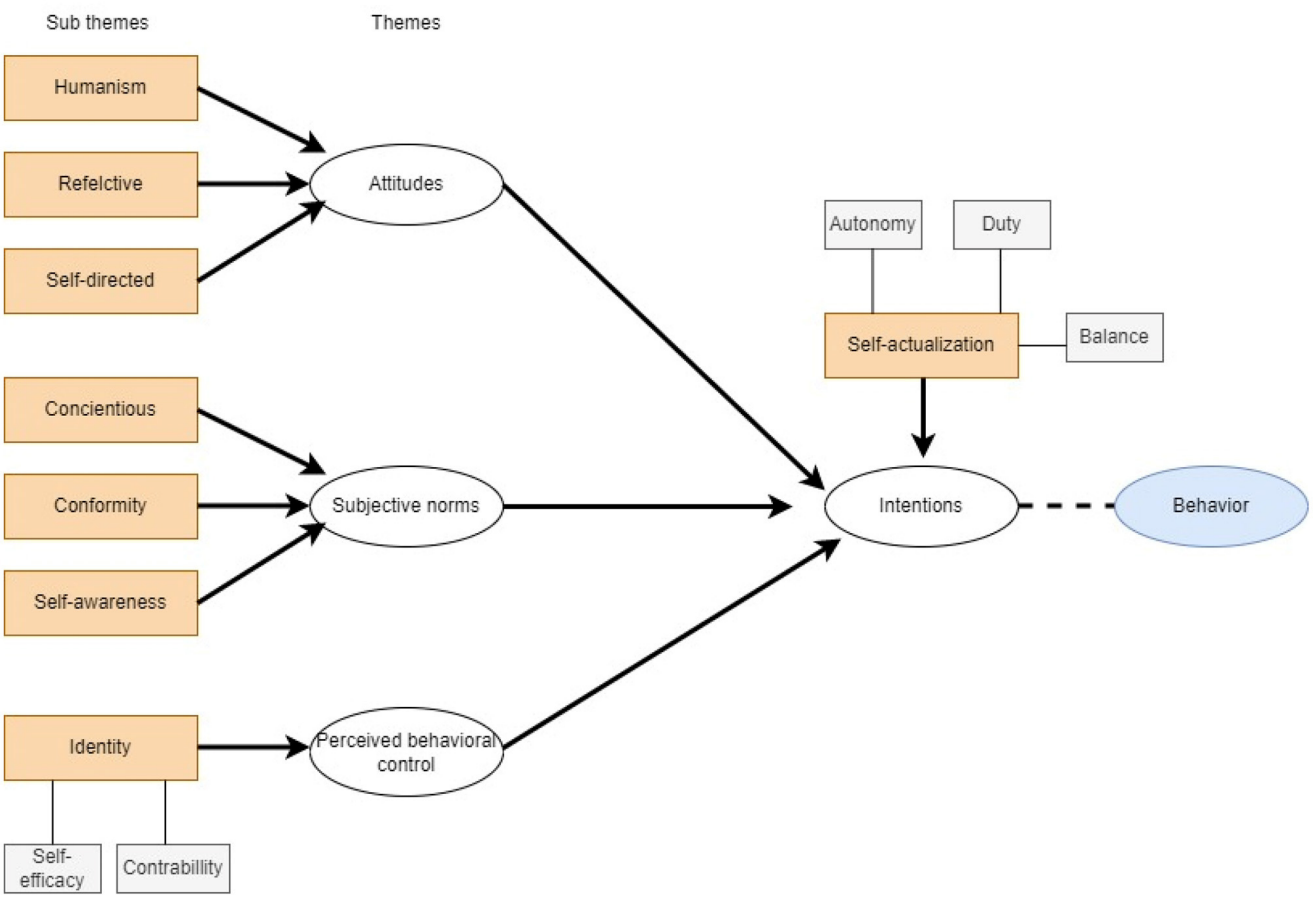
The process of thematic analysis of the qualitative data which depicts relationship of subthemes and themes. All themes essentially lead to possible change in an individual's behavior.

According to our suggested propositions of TPB and MEeP framework, our thematic analysis yielded four overarching theme their subthemes. The process of thematic analysis of the qualitative data which depicts relationship of subthemes and themes is shown in Figure 4. This intertwining of theoretical and professional frameworks generated a broad canvas of findings as detailed below. The below mentioned part of the manuscript provides a detail of themes along with the most relevant excerpts and their interpretations.

#### Theme I—Attitudes

Attitude constitutes a crucial determinant to affect behavioral intentions in TPB. If an individual likes a specific behavior, then the intention to conduct such behavior will be high. We found three subthemes: reflective, self-directed and humanism and most participants showed positive and favorable attitudes toward e-professionalism. The most common subtheme that emerged was being reflective. When asked about the freedom of speech, the participants showed a reflective attitude.

*-“If exercising your free speech could go against this code of conduct given by the institution/hospital you should reflect and re-consider your purposes/goals and behave in an ethical manner”- (Preclinical /RCSI)*

*-“Use your freedom of speech wisely and stay mindful of what you say as something which may seem respectful to you could hurt someone”- (Clinical /RCSI)*

Participants also showed a good understanding of the implications of befriending a patient on social media and how online communication was different from face-to face.

*-“Don't add your patients on social media. You're providing care, you're not there to be their friend. Forming one connection with a patient might open opportunities for more connections”- (Clinical/RCSI)*

*-“The idea of understanding between the poster and reader might be different. It will be the same but have a certain difference that we need to acknowledge such as nonverbal and the other person limitation in bringing the discussion”- (Clinical/USIM)*

Participants also highlighted the importance of keeping a check on their social media posts and online activities. The digital world being devoid of any need for a referee, made the idea of agency and constant self-checking essential determinants in this situation.

*-“You also need self-reflection, because although the physician may have posted something in the past, they still need to reflect back and like we all as physicians should reflect back on what we what we have been doing in the past, because it may reflect the image in the present”-(Clinical/UoS)*

The concept of agency was prominent in the participants' attitudes as they had a clear understanding of the status that healthcare professionals enjoy in society.

*-“Respect the opportunity, privilege and power given to medical students/professional”-(Preclinical/RCSI)*

*-“Physicians have a greater responsibility to get social media skills right and therefore, medical students should begin practicing proper conduct and take it with seriousness”-(Preclinical/USIM)*

A humanistic attitude was identified where participants emphasized the importance of factual accuracy of information posted online. Exponential increase of knowledge has placed another obligation on the users to maintain the information ecosystem for the betterment of society. This humanistic aspect of medicine with such a positive attitude in the digital world was evident among our participants.

*-“Use the social media to contribute the community in the way we spreading the awareness must be universalism so everyone has the right to get treatment and services from the provided doctor”-(Clinical/USIM)*

*-“Reflect and think about the patients' wellbeing and the necessity of sharing the information before doing so”-(Preclinical/RCSI)*

#### Theme II—Subjective Norms

Subjective norm explains an individual's understanding that most people who are important to him consider that he should or should not perform a behavior. Three subthemes were identified: *conscientiousness, self-awareness and conformity* which explain how these qualities shape the influence of subjective norms. Conscientiousness and self-awareness were dominant traits which described students' perceptions of the people who were essential to them. Once participants understood the overarching concept of mission, they realized that keeping a clean digital track record was as important as knowledge about social media account settings. This reflected their sense of belongings to the profession.

*-“Instead of just fixing your privacy settings, consider removing posts which may be conflicting to avoid future disputes and keep your reputation clean”- (Clinical/RCSI)*

*-“Depending on the patient and their values, different things can be a trigger. We should be considerate to the country we live in and the culture there”-(Clinical/UoS)*

*-“Medical students are held up to a high standard and their behaviours on social media directly reflects on the type of students the medical school grows”-(Clinical/USIM)*

When students grasped the understanding of their mission to be digitally aware, it was evident that they felt the pressure to behave, depict and represent a certain sector of society. It was natural for the individuals to modify their understandings and their online conduct if it was contrary to the group they belonged to. We noticed that when students were given the option to choose a personal vs. professional identity in the digital world, subjective norms played an important role in the real depiction of their identity and a confirmative approach.

*-“We carry the institution image - Healthcare professionals should be mindful that they are ambassadors of their institution. Their behaviour reflects not only on themselves but their place of employment”-(Clinical/UoS)*

*-“We represent a trustful group of society whom we feel safe with. So our picture should be appropriate”-(Clinical/UoS)*

#### Theme III—Perceived Behavior Controls

Perceived behavioral control is considered to determine behavioral intentions of individuals. Only one subtheme was identified under this main theme. We looked at the data from a self-efficacious and perceived controllability aspect. These two aspects made up the subtheme, identity. We identified the self-efficacy feature of identity as perceived behavioral control which was influenced by recognition of attitude and a control emanating from internal concerns. Such salient internal concerns to one's self concept shape self-identity. Participants showed a clear understanding of communication in the digital world. This self-efficacy was enhanced when clear institutional guidelines and policies were made available to the participants.

*-“Communication becomes risky when it's online because there's a possibility of that information becoming public”-(Preclinical/RCSI)*

*-“Rules need to be set and explained clearly to medical professionals in order to have the freedom and save themselves from serious consequences”-(Clinical/RCSI)*

We sensed that an understanding of true meaning of their identity was an effective driver in the digital world which allowed them to adjust their internal locus of control. Digital world being public, powerful, and permanent exerted that power by which self-regulation shaped the participant's identity. But an inability to maintain that control was also attributed to a peculiar nature of online world.

*-“Yes, however it is increasingly hard since the digital footprint you leave behind can never be erased and it is easier not posting than having to deal with things you post in the future. It's possible if you really limit posting on social media, thereby decreasing your digital footprint”-(Preclinical/RCSI)*

*-“We are much more careful about what we say in real life but when its online, which is impulsive, easy going and nontolerant, we tend to forget that we are breaching others privacy”-(Clinical/RCSI)*

*-“Often people have more confidence over social media because they are hiding behind a screen, so they may be more outspoken on social media”-(Preclinical/USIM)*

An interesting aspect emerged where participants agreed that digital world has led to a context collapse and a clear distinction of personal and professional self was difficult to obtain. This strengthened their self-efficacy to manage their online identities in a conformative manner.

*-“It is close to impossible to separate your personal and professional life online and so due to this, i think it is best to be aware of what you post and think about how that may affect your professional life while still maintaining a private life, yes this is limiting and it is not sustainable, but with today's digital world, there is very little you can hide from the public”-(Preclinical/RCSI)*

Our participants recognized the concept of volitional control, controllability of their image and self-identity. This aspect showed their motivation and their power to control self-image (personal vs. professional). It was also evident that the understanding of mission and a sense of belonging to a particular sector allowed to think them beyond this personal vs. professional self and empowered the participants to shift locus of control in the right direction.

*-“Remember you are a healthcare professional and remember your boundaries”-(Clinical/RCSI)*

Although, external control was rightly understood but still some participants argued that high expectations placed on them as healthcare professionals by the society was not always right.

*-“Judgements are often made about health professionals competence based on their profile, which is not always correct but understandable”-(Clinical/USIM)*

*-“We represent a trustful group of society whom people feel safe with. So our picture should be appropriate”-(Clinical/UoS)*

These findings highlight a general sense of motivation which could be considered as an antecedent of intention. However, there were some remarks which gave insights into the frustration of participants due to societal expectations and how society judge them, indicating an obvious gap between perceived behavioral control and intentions. This brought a tension in the seamless integration of the digitally natives subjects between the online and physical realms. This strengthens the assumption that several behaviors were not completely under one's volitional control. This was more prevalent in preclinical students who struggled to identify a clear line between professional and personal territories.

*-“I don't get the point, just coz we are medical students why should we have a professional looking social media account, isn't fun not allowed? “But this also brings conformity into question. A lot of people will post of social media to fit in with others and be part of a society (e.g., flex culture).”-(Preclinical/RCSI)*

*-“The problem is related to the expectations that the society had set to doctors. We all had lives before becoming doctors, its insane to judge someone for something social media post as such, it just seems really odd to base her judgement on a picture of him rather than his actual practice”-(Preclinical/UoS)*

*-“It's unfair to assume that doctors make no mistakes whatsoever. We are all humans but on the other hand some doctors also have a god complex”-(Preclinical/USIM)*

#### Theme IV—Intentions

Intention discerns the extent to which a individual intends to carry out the behavior in future. Self-actualization was the main subtheme under intentions. Self-actualization is the ultimate need and one of the motivating elements in the realization of one's own maximum potential. A unique finding from the inductive analysis yielded the process of achieving one's own full potential through balance, duty, integrity, autonomy, and respect of one's desires and wishes, the core tenets of medical profession that help to fulfil the professional responsibilities.

*-“Keep a balance. Stay alert and be focused. Stay true to yourself and your values and be mindful of what your words can do to others be beyond the muddle you can create a public page to share, and you can have a personal page for your leisure time without the public pressure.”-(Clinical/UoS)*

*-“We have to self-direct ourselves and think about the cause and effect of my action”-(Clinical/USIM)*

*-“Reflection, accountability and duty should be taken into professional connection in the digital world”-(Clinical/USIM)*

We also noticed how the participants understanding of the “self” evolved and brought forward the concept of singularity of the “digital self” when the mission was understood.

*-“The sooner we realize our mission, our status, the faster we can differentiate what we need to do and avoid in this digital world”-(Clinical/UoS)*

### TPB Factors and MEeP Constructs—Data Triangulation

Combining the results from quantitative (survey) with qualitative (Jamboard contributions and breakout rooms recordings) we identified some congruence as well as some significant dissimilarities. Participants who attended the workshop showed a significant improvement in attitudes and subjective norms leading to increased chances of intentions to be digitally professional in the values, behaviors and identity constructs depicted by the path analysis. However, perceived behavioral control did not show a significant positive relationship with the intention. Same pattern crystallized in qualitative findings where perceived behavioral control emerged as identity (self-efficacy and perceived controllability) lacked a control emanating as internal concerns. These concerns were raised due to lack of concrete guidelines and the distinct nature of digital world. Participants asked for ever dynamic social media specific institutional guidelines. While manipulative, intrusive, and uninhibited nature of digital world makes it difficult to regulate internal locus of control. However, some excerpts spoke loudly than rest about the clear understanding of mission. This aspect also strengthens the Cruess (35) take on professional identity formation. Peripheral participation in community of practices will not strengthen the perceived behavioral control as the subjective norms are not truly understood by the individuals which was a striking dissimilarity in quant-qual results. Intentions will improve only once the self-actualization sets in. For this to ensue, core attributes of MEep framework (integrity, benevolence, tolerance and self-directions) are required. This post-positivist approach strengthened our analyses by mapping the relation from different points of reference.

## Discussion

Our mixed method study has evaluated the MEeP framework with its mission-based constructs of professional values, behaviors and identities using the theoretical underpinning of TPB as a benchmark. The interventional workshop using MEeP framework improved attitudes, subjective norms, perceived behavioral control, and intentions. The quantitative data highlighted the participants' intentions to be digitally professional with an improvement in attitude in terms of value (whistleblowing-raising concerns), behavior (being responsible in digital world) and identity (being reflective in digital world) constructs. A significant relationship was found between attitudes and subjective norms, and perceived behavioral control, but a gap was evident when perceived behavioral control and subjective norms couldn't alter their intentions to be digitally professional. This finding was also reflected in the qualitative data where participants showed a positive attitude by showing reflective, self-directed, and humane attributes. Conscientiousness, self-awareness and conformative aptitudes were shaped by subjective norms, while identity formation, controllability and self-efficacy values fell short of achieving self-actualization.

The forthcoming section of the discussion elaborates the key findings of our research with reference to four elements of TPB.

### Attitudes

Evidence-based research has eluded that individuals show positive attitudes toward certain behaviors when they strongly perceive a positive outcome by adopting that behavior (36). Likewise, our study has reported a positive impact of attitudes on behavioral intentions to use the social media related services. We have also noticed that the participants believed in reflection as the first step toward the professional identity formation. Reflection is considered as the process of analytical thinking by individuals which may be potentially relatable to their professional practice (37). In our study, we found reflective, self-direction and humanism as three major concepts under professional attitudes. *Reflective* approach is a powerful human characteristic which enriches individuals in appraising and reflecting on their own experiences (38). William Branch has rightly argued that longitudinal educational pedagogies of critical reflection tend to enhance humanistic values with substantial transformative impact on learners (39). Owing to the fluid nature of professional values, behaviors and identities in the digital realm, a robust reflective practice would help steer the HCPs and medical students to develop their own appetite toward e-professionalism. This is a positive finding from our research which articulates well with the basic principles of professional conduct in the digital climate (40).

*Self-direction*, another fundamental pillar of autonomy, relies on self-regulation (41). Self-direction is closely related to self-actualization, defined by Maslow as “using one's capabilities in the most creative and effective way” (41). Interestingly, the digital world has no reference, and this pitfall intensifies the need of an agency for professional diligence (42, 43). Our interventional workshop underpinned the significance of giving priority to the participants' ability to act as agency. Such empowerment brought context-based results which are extrapolated toward self-efficacy and self-actualization. Our participants preferred self-regulation and self-actualization to empower their beliefs and potentials for managing challenges and obstacles in achieving their targets.

Recently, we have witnessed an exponential growth of the digital contents of biomedical knowledge. HCPs, physicians and medical students have now a generic trend to update, and not to recall, biomedical knowledge from an ubiquitous and digitalized health care repositories (44). Unfortunately, the Internet and especially social media contain a mix of concrete evidence based scientific knowledge as well as unverifiable information. This creates an uncertainty about the accuracy of digital contents which leads to misleading information with its potential adverse consequences (45). As evident in our research, most participants preferred *humanism*, another constituent of attitudes, which may allow them to analyse Internet-based biomedical information using a personalized humanistic perspective. Therefore, a humanistic factor, along with a technology-based approach is essential in accessing accurate biomedical information (46).

### Subjective Norms

Subjective norms, “the degree of pressure felt from various aspects to act in a desired manner,” can be conveniently divided into two categories: subjective injunctive norm (individual's perceptions) and subjective descriptive norm (behaviors elicited in a social environment) (47). According to Ajzen, in TPB, subjective norms play a pivotal role in altering human behaviors and attitudes (48). In addition, subjective norms can be determined by normative beliefs and by the motivation to accept certain beliefs. Therefore, subjective norms can be related to social identity (49). Our study illustrated a strongly positive correlation between subjective norms and intentions, but only for participants who were willing to develop strong professional relations with peers.

Our research has also demonstrated that subjective norms are substantially associated with the referents' behavioral intentions toward being digitally expert. Such referents include friends, families, and peers. Generally, individuals are keen to get acquaintance with their referents (50). However, in our study, the participants were unwilling to own a problem due to the perceived associated adverse consequences of increased workload and due to the uncertainty about the existing regulations about the usage of social media. This finding underpins the need for an emphasis on organizational as well as subjective norms in making the individuals aware of personal and professional ownership of roles and responsibilities. Under subjective norms, we found three subthemes of *conscientiousness, self-awareness*, and *conformity*. *Conscientiousness* and *self-awareness* encompass personality traits that reflect students' perceptions of the people whom they consider to perform a behavior (51). In our study, the participants considered conscientiousness and self-awareness as sources of inspiration and commitment in decision-making, judgements, and interpersonal engagements.

*Conformity* pertains to a specific internalized and personalized impression which may include a glimpse of ethical, professional or collegial (52). In an interesting study by Beran et al., the authors have demonstrated that the learners, while performing knee arthrocentesis, would insert a needle into an wrong anatomical location if they had strong reasons to believe that their peers had also inserted a needle into the same site (52). Such phenomenon of conformity reflects the influence of peers and referents on one's performance, behavior, and conduct. Our study has also depicted the impact of conformity as most participants did not want to stand out of the peer group due to either being more compliant to the group or due to the lack of confidence in asserting their opinions during group discussions.

### Perceived Behavioral Control

Perceived behavioral controls are the individuals' ability belief to carry out a behavior (53). Under perceived behavioral controls, our study identified *self-efficacy* (pertaining to the ease or difficulty of carrying out a behavior) and *perceived controllability* (the degree to which behavioral conduct depends upon the person performing that behavior). Both subthemes heavily rely on an easy access to the resources essential to perform certain behaviors (54). Self-efficacy and perceived controllability by the participants in our study led them to endorse a crucial role of HCPs and medical students in defining their own digital space in social media. This reflects an appraisal of their self-confidence and the ease of control over their behaviors. Such phenomenon resonates well with the finding that digitally native users of the Internet and social media are self-efficacious and possess a better control over their drive to use technology-based tools in the medical field (55). However, most participants agreed that regulatory guidance on raising a concern would have given them a contextualized support in the digital environment and will increase their self-efficacy.

### Intentions

Under intentions, we identified *self-actualization* subtheme which sheds light on subjective experience with a discourse to find out one's own potential (56). Self-actualization contains one's emotions, empathy, interpersonal relations, and the ability to express one's influences and cognitive knowledge (57). From the context of HCPs in the digital realm, medical educators aim at the psychological, professional, and technical profiling of self-actualized medical personnel (58). In our study, we noticed that the participants recognized that self-understanding of their objective sense of digital reality, accepting their own experiences, autonomy and mission had a significant impact on the case scenario analysis.

### Study Limitations

Our mixed method study had a small sample size. However, from the viewpoint of a focused and precise aim of the study, a specific sample worked well on the application of an established TPB. Also, data triangulation across methods required matching participants across qaun-qaul designs hence our study ended up in a smaller sample size (59). This brought integrated and intense discussions among the participants and facilitators. Another weakness of our study was the representation the workshop facilitators from a diverse range of clinical and basic sciences in the medical and dental disciplines. While this can be viewed as a strength, a homogenized group of facilitators would have given a more contextualized data. Nevertheless, the reported data meticulously fulfills our research requirements, and this has enabled us to address all research questions. Lastly, an absence of the participants' previous knowledge of e-professionalism was evident. We circumvented this shortcoming by providing pre-recorded lecture on a digital platform, a comprehensive participant information toolkit, and a facilitator guide. This managed to harmonize the knowledge and approach of all stakeholders toward e-professionalism before and during the workshop.

## Conclusion

This mixed method study has diligently evaluated the MEeP framework using the elements of TPB as a benchmark. The study identified significant improvement in attitudes, subjective norms, perceived behavioral control, and intentions in the pre-post analysis. There was significantly positive relationship between attitudes and subjective norms, attitudes and perceived behavioral control. In contrast, perceived behavioral control and subjective norms failed to change the participants' intentions to be digitally professional. Mostly, there was a positive attitude toward reflection, self-direction, and humane attributes. Though the values of conscientiousness, self-awareness and being conformative were enhanced by subjective norms, the identity formation, controllability, and self-efficacy values could not achieve self-actualization. Our study endorses a successful application of the mission based MEeP framework in enhancing the professional values, behaviors, and identities of undergraduate medical and dentals students.

## Data Availability Statement

The raw data supporting the conclusions of this article will be made available by the authors, without undue reservation.

## Ethics Statement

The studies involving human participants were reviewed and approved by RCSI-MUB REC 139 / 25-Mar-2021 University of Sharjah REC-21-06-03-01 University Sains Malaysia Research Ethics Committee (JEPeM) USM/JEPeM/19050291. The patients/participants provided their written informed consent to participate in this study.

## Author Contributions

SSG conceived and drafted the idea, prepared the proposal, developed the content, prepared the relevant appendices, conducted the facilitators training session,and applied for ethical approvals. SSG, FR-D, MY, and SYG contributed substantially to the development of workshop plan. SSG and MH analysed the data and prepared the results. Final draft was prepared by SSG while SYG and FR-D proofread and improved the intellectual content. FR-D, SYG, SF, DH, and MY reviewed the first and final draft. All members underwent multiple and iterative training sessions to unify the workshop process and agreed to take responsibility for the final draft submission. All authors contributed to the article and approved the submitted version.

## Funding

This research was funded by The School of Postgraduate Studies and Research of The Royal College of Surgeons Ireland – Bahrain Medical University Bahrain.

## Conflict of Interest

The authors declare that the research was conducted in the absence of any commercial or financial relationships that could be construed as a potential conflict of interest.

## Publisher's Note

All claims expressed in this article are solely those of the authors and do not necessarily represent those of their affiliated organizations, or those of the publisher, the editors and the reviewers. Any product that may be evaluated in this article, or claim that may be made by its manufacturer, is not guaranteed or endorsed by the publisher.
