# Supplementary material for: Changing Professional Behaviors in the Digital World Using the Medical Education e-Professionalism (MEeP) Framework—A Mixed Methods Multicentre Study
Source: Front Med (Lausanne). 2022 Mar 28;9:846971. doi: 10.3389/fmed.2022.846971 (PMC9004460; doi:10.3389/fmed.2022.846971)
Supplement: Supplementary file 1 [file Data_Sheet_1.PDF]

Appendix I Brief Plan of Workshop

Workshop Goal and Objectives
Goal:
Participants will articulate the elements of e-professionalism and apply them to curated sets of scenarios.

Objectives:
At the end of the workshop, learners will be able to remember ***DAGaRR:***
1. Define the terms and features associated with the concept of e-professionalism at an abstract level and concrete, i.e., behavioral, level.
2. Acquire experience in defining and articulating professional and unprofessional behaviors by describing various behaviors related to a common set of experiences illustrated in the scenarios.
3. Gain perspectives on how the same experience can be perceived from multiple perspectives of other individuals on a team (e.g., student, resident, faculty, family, society and council, etc).
4. Recognize behaviors in yourselves and others that can be categorized using the elements of e-professionalism.
5. Reflect on the workshop experience in terms of your behavior and that of others related to e-professionalism

Time	Activities	Facilitator(s)	Comments
5 minutes	Introduction<br>• Goal & objectives<br>• Structure		
5 minutes	Allotment of breakout rooms and jam board links in the chat		
50 minutes	Breakout rooms<br>• 40 minutes for case studies<br>• 10 minutes for presentation<br>Detailed guide and facilitator toolkit provided		
40 minutes	Large group session<br>10 groups x 3 minutes each<br>10 minutes for wrap up.		
10 minutes	Post workshop Survey		
