# Supplementary material for: Changing Professional Behaviors in the Digital World Using the Medical Education e-Professionalism (MEeP) Framework—A Mixed Methods Multicentre Study
Source: Front Med (Lausanne). 2022 Mar 28;9:846971. doi: 10.3389/fmed.2022.846971 (PMC9004460; doi:10.3389/fmed.2022.846971)
Supplement: Supplementary file 2 [file Data_Sheet_2.PDF]

Appendix II – Theory of Planned Behaviour Questionnaire

Exploring Medical students' Behaviours about e-professionalism

I. Whistleblowing – Raising concern

The following questions are about raising a concern at your academic institution. We would like toknow what you think about this, regardless of whether or not you have had/seen a significant issue at your school/place/institution e.g. a post breaching the code of social media conduct of your institution.

1. Overall, I think that raising a concern is: *(please circle)*

Worthless	1	2	3	4	5	6	7	***Worthwhile***
Unpleasant	1	2	3	4	5	6	7	***Pleasant***
Difficult	1	2	3	4	5	6	7	***Easy***
The wrong thing to do	1	2	3	4	5	6	7	***The right thing to do***
Bad practice	1	2	3	4	5	6	7	***Best practice***

2. Please rate how much you personally agree or disagree with the statements below: *(please circle)*

	Strongly disagree						***Strongly agree***
**i. I would feel comfortable speaking to the concerned department if I had a concern at my college**	1	2	3	4	5	6	7
**ii. People who are important to me think thatI should not report a concern if I have one (R)**	1	2	3	4	5	6	7
**iii. I plan to raise a concern if I have one in my school**	1	2	3	4	5	6	7
**iv. I don't intend to raise a concern if I have one in my school (R)**	1	2	3	4	5	6	7
**v. It is expected of me that I report a concern if I have one**	1	2	3	4	5	6	7
**vi. I am confident that I can raise a concern if I want to**	1	2	3	4	5	6	7
**vii. I want to raise a concern when I have one in my school**	1	2	3	4	5	6	7

3. Please indicate how much pressure you would feel from each of the following organisations or people to raise a concern if you had one *(please circle)*:

	No pressure						***Strong pressure***
**i. My institution**	1	2	3	4	5	6	7
**ii. Myself/My Trust**	1	2	3	4	5	6	7
**iii. Medical Council**	1	2	3	4	5	6	7
**iv. Personal tutor/supervisor**	1	2	3	4	5	6	7
**v. Peers**	1	2	3	4	5	6	7
**vi. Teachers**	1	2	3	4	5	6	7
**vii. Society**	1	2	3	4	5	6	7
**viii. Patients**	1	2	3	4	5	6	7
**ix. The media**	1	2	3	4	5	6	7

4. For me to report a patient safety concern is: *(please circle)*

Difficult	1	2	3	4	5	6	7	***Easy***

Attitudes: 1.1-1.5 + 2.1;
Subjective norms: 2.2 + 2.5 + 3.1-3.9;
Perceived behaviours control: 2.6 + 4;
Intentions: 2.3 + 2.4 + 2.7

II. Reflective Practice

5. Overall, I think that reflective practice on my social media activity is:

Worthless	1	2	3	4	5	6	7	***Worthwhile***
Difficult	1	2	3	4	5	6	7	***Easy***
Irrelevant	1	2	3	4	5	6	7	***Relevant***
Unpleasant (for me)	1	2	3	4	5	6	7	***Pleasant (for me)***
The wrong thing to do	1	2	3	4	5	6	7	***The right thing to do***
Bad practice	1	2	3	4	5	6	7	***Best practice***

6. Please rate how much you personally agree or disagree with the statements below:

	Strongly disagree					***Strongly agree***	
**i. Reflecting on my social media practices makes me a better medical student**	1	2	3	4	5	6	7
**ii. Reflecting on my social media practices demonstrates that I am trying to become a better medical student**	1	2	3	4	5	6	7
**iii. I do not plan to reflect on my social media practices (R)**	1	2	3	4	5	6	7
**iv. People who are important to me think thatI should reflect on my social media practices**	1	2	3	4	5	6	7
**v. I intend to reflect on my social media practices**	1	2	3	4	5	6	7
**vi. It is expected of me that I reflect on my social media practices**	1	2	3	4	5	6	7
**vii. I want to reflect on my social media practices**	1	2	3	4	5	6	7

7. Please indicate how much pressure you feel from each of the following organisations or people to reflect on your practice:

	No pressure				***Strong pressure***		
**i. My institution**	1	2	3	4	5	6	7
**ii. Myself/My Trust**	1	2	3	4	5	6	7
**iii. Medical Council**	1	2	3	4	5	6	7
**iv. Personal tutor/supervisor**	1	2	3	4	5	6	7
**v. Peers**	1	2	3	4	5	6	7
**vi. Teachers**	1	2	3	4	5	6	7
**vii. Society**	1	2	3	4	5	6	7
**viii. Patients**	1	2	3	4	5	6	7
**ix. The media**	1	2	3	4	5	6	7
**x. Healthcare professionals**	1	2	3	4	5	6	7

8. For me to reflect on my practice is: *(please circle)*

Difficult	1	2	3	4	5	6	7	***Easy***

Attitudes: 5.1-5.6 + 6.1 + 6.2;
Subjective norms: 6.4 + 6.6 + 7.1-7.10;
Perceived behaviours control: 8;
Intentions: 6.3 + 6.5 + 6.7

III. Responsible in digital world

The following questions are about the responsible behaviour expected of a medical student in anonline world.

9. Overall, I think that being responsible while using social media is:

Difficult	1	2	3	4	5	6	7	***Easy***
Irrelevant	1	2	3	4	5	6	7	***Relevant***
Unrealistic	1	2	3	4	5	6	7	***Realistic***
Unclear	1	2	3	4	5	6	7	***Clear***
Harmful	1	2	3	4	5	6	7	***Beneficial***
Worthless	1	2	3	4	5	6	7	***Worthwhile***

10. Please rate how much you personally agree or disagree with the statements below: *(please circle)*

	Strongly disagree					***Strongly agree***	
**i. People who are important to me think I behave responsibly while sharing content on social media**	1	2	3	4	5	6	7
**ii. It is expected of me to be respectful of conflicting opinions in the digital world**	1	2	3	4	5	6	7
**iii. I am more likely to check the privacy and confidentiality issues of any content I am posting *(R)***	1	2	3	4	5	6	7
**iv. I am more likely to remove any contradictory post from my social media account *(R)***	1	2	3	4	5	6	7
**v. I am confident that I can apply the social media code of conduct**	1	2	3	4	5	6	7
**vi. I have enough time to refer to the my institution social media guidance**	1	2	3	4	5	6	7
**vii. I can easily discern the right and wrong in digital world with a desired level of evidence**	1	2	3	4	5	6	7
**viii. I intend to maintain a professional relationship with my peers and co-workers in online world**	1	2	3	4	5	6	7
**ix. I want to use the right privacy settings on social media account**	1	2	3	4	5	6	7
**x. I do not plan to be responsible in online world (R)**	1	2	3	4	5	6	7

11.Please indicate how much pressure you feel from each of the following organisations or people to use the GMC's confidentiality guidance: *(please circle)*

	No pressure				***Strong pressure***		
**i. My institution**	1	2	3	4	5	6	7
**ii. Myself/My Trust**	1	2	3	4	5	6	7
**iii. Medical Council**	1	2	3	4	5	6	7
**iv. Personal tutor**	1	2	3	4	5	6	7
**v. Peers**	1	2	3	4	5	6	7
**vi. Teachers**	1	2	3	4	5	6	7
**vii. Society**	1	2	3	4	5	6	7
**viii. Patients**	1	2	3	4	5	6	7
**ix. The media**	1	2	3	4	5	6	7

12.For me to apply the GMC confidentiality guidance in my practice is: *(please circle)*

Difficult	1	2	3	4	5	6	7	***Easy***

Attitudes: 9.1-9.6 + 10.3 + 10.4;
Subjective norms: 10.1 + 10.2 + 11.1 - 11.9;
Perceived behaviours control: 10.5 + 10.6 + 10.7 + 12;
Intentions: 10.8-10.10
