# Supplementary material for: Changing Professional Behaviors in the Digital World Using the Medical Education e-Professionalism (MEeP) Framework—A Mixed Methods Multicentre Study
Source: Front Med (Lausanne). 2022 Mar 28;9:846971. doi: 10.3389/fmed.2022.846971 (PMC9004460; doi:10.3389/fmed.2022.846971)
Supplement: Supplementary file 3 [file Data_Sheet_3.PDF]

**Appendix III FACILITATOR'S GUIDE**

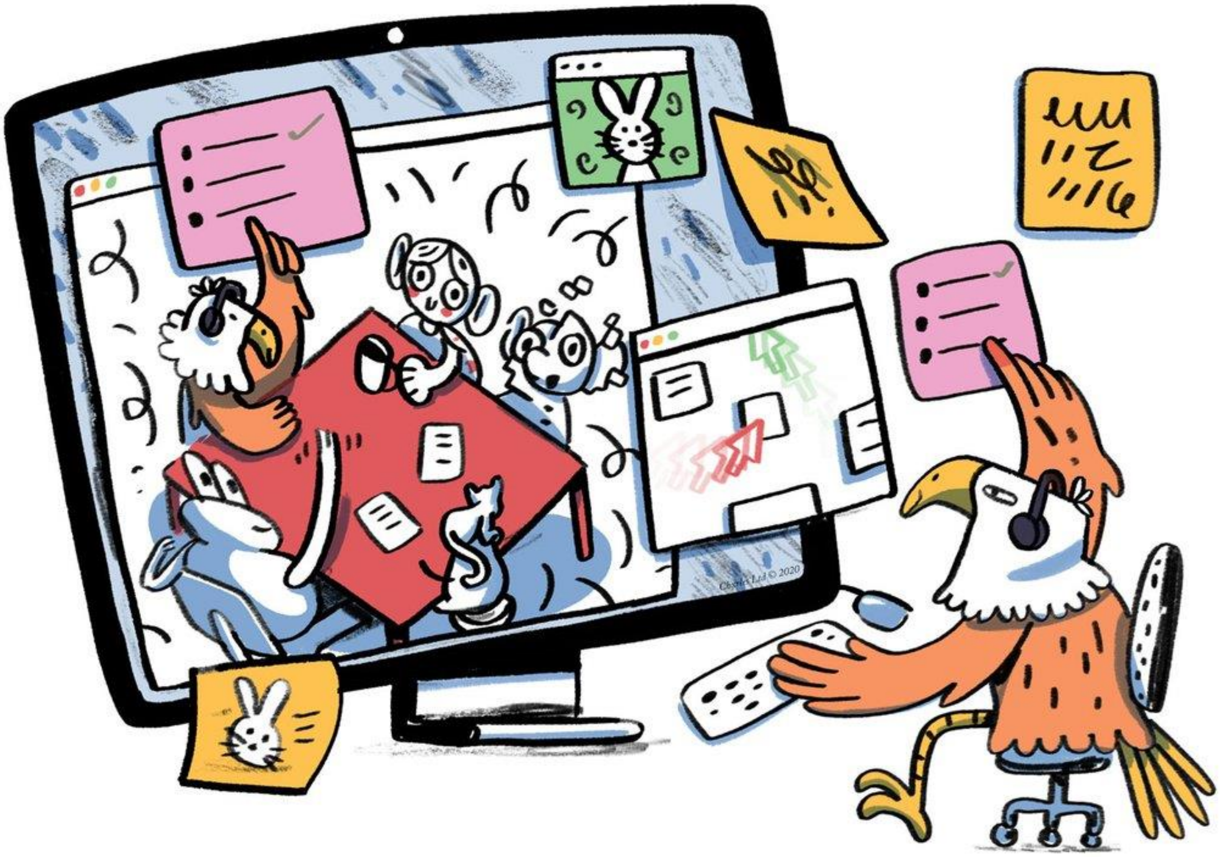

**Goal:** To apply the elements of e-professionalism to daily clinical practice.

At the end of the workshop, learners will be able to remember ***DAGaRR:***

**1. Define** the terms and features associated with the concept of e-professionalism at an abstract level and concrete, i.e., behavioral, level.

**2. Acquire experience** in defining and articulating professional and unprofessional behaviors by describing various behaviors related to a common set of experiences illustrated in the scenarios.

**3. Gain perspectives** on how the same experience can be perceived from multiple perspectives of other individuals on a team (e.g., student, resident, faculty, family, society and council, etc).

**4. Recognize** behaviors in yourselves and others that can be categorized using the elements of e-professionalism.

**5. Reflect** on the workshop experience in terms of your behavior and that of others related to e-professionalism.

**STEP BY STEP FACILITATOR'S GUIDE FOR SMALL GROUP DISCUSSION PORTION OF THE WORKSHOP**

1. **Introduce** yourself as the facilitator of the small group session.
2. E**xplain** the process of moving through at least the first 4 scenarios in 60 minutes and recording the participants' thoughts on the Jam board (which is like an electronic flip chart), you will have 10 minutes to discuss the scenario and questions posed and then a further 2 mins to assist the scribe to make notes for a presentation slide on each scenario.
3. In the breakout room your cameras should be **switched OFF.**
4. Microphones should be **turned ON when you want to speak** especially during the take home message part.
5. Remind participants that the sessions are **RECORDED.**
6. Remind the **participants that THE BREAKOUT ROOM is a SAFE SPACE all contributions** are anonymised to ensure that they will make **AUTHENTIC and HONEST contributions to the discussion**.
7. Invite one member of the group to be both the **SCRIBE AND SPOKEPERSON** their job is to make notes during the discussion of each scenario and then summarise this on the empty frame on the Jam board slides.
8. Remind them that the job of **SPOKESPERSON** is to also present their contribution later in the large group session
9. In the first question **participants will be asked to recall the MEeP framework** and to use the desired attributes in the four constructs: Values, Behavior, Identity and Mission.
10. **Have ONE Participant READ THE FIRST scenario:** Move through the questions ensuring that all have completed sticky notes, if there is hesitancy give them a few seconds and then prompt again to phrase the question a little differently.
11. **For "take home message"** encourage participants to verbalize their opinions to tease out a meaningful discussion.
12. **Before you leave the scenario to move to the next one, all the group should help the SPOKESPERSON to prepare a slide/frame for the LARGE GROUP DISCUSSION:** Review the themes that emerged during the small group discussion, or just reiterate what the group learned. This preparation will facilitate the role of the group's spokesperson during the large group wrap-up session.

**AT THE END OF THE BREAKOUT ROOM SESSION REMIND THE PARTICIPANTS TO COMPLETE THE POST WORKSHOP SURVEY!**
